# One more grammar for causality - understanding interventionism

**DOI:** 10.1192/j.eurpsy.2025.1940

**Published:** 2025-08-26

**Authors:** N. Ramalho, T. Rocha, J. F. Cunha, J. C. Moura, J. Leal, D. Seabra, I. Lopes, G. Santos, M. Rosa, A. Garcia

**Affiliations:** 1 Psychiatric and Mental Health Department, ULSAR, Barreiro, Portugal

## Abstract

**Introduction:**

The role of causality in reality has sparked a long and ongoing debate that began with Aristotle, but in its modern form, having reverberated to this day, found its origin in Hume. In psychiatry, causality gains additional layers since the domain of intelligibility does not extend as broadly as it does to the natural sciences, due to the nature of both its object and its method.

**Objectives:**

To explore proposals for understanding causality in psychiatry and mental illnesses.

**Methods:**

A non-systematic literature review was conducted using the PubMed/MEDLINE and PhilPapers databases with the search terms “causality,” “psychiatry,” “interventionism,” and “causal grammar.” Reference bibliography was also consulted.

**Results:**

Causal interventionism is a way of understanding causality, where performing an intervention in groups allows the distinction between causality and mere correlation, utilizing counterfactuals that are verified in light of that intervention. The idea of causal grammar allows causal explanation to be thought of as a matter of finding a family of interventions in variables that make a difference to the outcome variable, governed by the causal grammar of that domain.

**Conclusions:**

While each of the positions addresses certain issues—interventionism tackling various levels of explanation of causality in psychiatry, whether biological or psychodynamic, and causal grammar seemingly overcoming mechanismism—neither is fully satisfactory: in interventionism, the unfolding of causes, and in causal grammar, the pre-theoretical intuition seems challenged.

**Disclosure of Interest:**

None Declared

